# Synergistic effect of human CycT1 and CRM1 on HIV-1 propagation in rat T cells and macrophages

**DOI:** 10.1186/1742-4690-6-43

**Published:** 2009-05-12

**Authors:** Hiroyuki Okada, Xianfeng Zhang, Ismael Ben Fofana, Mika Nagai, Hajime Suzuki, Takashi Ohashi, Hisatoshi Shida

**Affiliations:** 1 Institute for Genetic Medicine, Hokkaido University, Kita-ku, Sapporo 060-0815, Japan; 2 Microbiology Division, New England Primate Research Center, Harvard Medical School, One Pine Hill Drive, Southborough, Maryland 01772, USA

## Abstract

**Background:**

*In vivo *studies of HIV-1 pathogenesis and testing of antiviral strategies have been hampered by the lack of an immunocompetent small animal model that is highly susceptible to HIV-1 infection. Although transgenic rats that express the HIV-1 receptor complex hCD4 and hCCR5 are susceptible to infection, HIV-1 replicates very poorly in these animals. To demonstrate the molecular basis for developing a better rat model for HIV-1 infection, we evaluated the effect of human CyclinT1 (hCycT1) and CRM1 (hCRM1) on Gag p24 production in rat T cells and macrophages using both established cell lines and primary cells prepared from hCycT1/hCRM1 transgenic rats.

**Results:**

Expression of hCycT1 augmented Gag production 20–50 fold in rat T cells, but had little effect in macrophages. Expression of hCRM1 enhanced Gag production 10–15 fold in macrophages, but only marginally in T cells. Expression of both factors synergistically enhanced p24 production to levels approximately 10–40% of those detected in human cells. R5 viruses produced in rat T cells and macrophages were fully infectious.

**Conclusion:**

The expression of both hCycT1 and hCRM1 appears to be fundamental to developing a rat model that supports robust propagation of HIV-1.

## Background

A small-animal model of HIV-1 infection is needed for development of prophylactic vaccines and more efficient antiviral therapies. Current animal models of HIV infection, including non-human primates [1-4] and severe combined immunodeficiency (SCID) mice transplanted with fetal human cells [5,6], have made significant contributions to our understanding of lentiviral pathogenesis and to the development of vaccines and therapeutic agents. However, these models have shortcomings, such as their limited availability and high cost, their permissivity restricted to related retroviruses of nonhuman primates, as well as the absence or insufficient induction of an immune response against HIV-1. Therefore, a better small-animal model is needed.

Rodents may be useful models if they can be infected with HIV-1. Because they are established experimental animals, inbred strains are available, and genetic manipulations can be performed. However, a fully permissive model has not been developed yet because of several inherent blocks to HIV-1 replication in rodent cells. One major block to HIV-1 replication is at the level of viral entry into the cell; this may be overcome by introducing human CD4 (hCD4) and CCR5 (hCCR5) [7,8]. Indeed, transgenic (Tg) rats expressing these receptors support some HIV-1 replication, albeit poorly [8], whereas Tg mice expressing hCD4 and hCCR5 do not support any HIV replication [9]. These results suggest that rats may provide a good small-animal model.

Studies on rodent cell-specific defects in the HIV-1 life cycle after viral entry provide the molecular basis for improving the propagation of HIV in rodents. However, several studies using established cells lines [7,10,11] have indicated that there are cell line specific defects in each step of the viral life cycle. Moreover, technical difficulties have hampered detailed analyses of the function of cellular cofactors in rodent T cells and macrophages, particularly primary cells.

A study of the effects of rodent cellular factors on the function of the viral factors Tat and Rev will be of importance because of the essential roles these proteins play in viral propagation. Currently, controversial results have been reported regarding the existence of a profound block affecting Tat function in rodent cells. In early studies, human CyclinT1 (hCycT1), identified as a Tat interacting protein that is crucial for transcription during HIV-1 replication [12], was expressed in mouse NIH 3T3 fibroblasts and transcriptional activity was dramatically enhanced [13,14]. Moreover, hCycT1 Tg mice supported the enhanced expression of an integrated HIV-1 provirus [15]. A single amino acid difference between human and mouse CyclinT1 (mCycT1), which has a tyrosine at residue 261 in place of the cysteine amino acid in hCycT1, causes almost a complete loss of Tat cofactor activity [13,14]. In contrast to mouse cells, rat cells support significant amounts of Tat function, even though rat CyclinT1 (rCycT1) has a tyrosine at residue 261 and shares ~96% sequence homology with mCycT1. Only 2–5 fold enhancement of Tat function by overexpression of hCycT1 in rat cells has been reported. Moreover, since the reported experiments lacked the expression of rCycT1 as a control, uncertainty remains whether it was the quantity or the quality of exogenously-expressed hCycT1 which augmented Tat function [7,16,17]. On the other hand, a substantial increase in Gag protein levels upon hCycT1 expression in a rat myelomonocytic precursor cell line has been reported [18].

Rev function is involved in the expression of the unspliced 9-Kb and partially-spliced 4-Kb RNAs that encode the HIV viral genome and the structural proteins [19]. Rev activity that supports HIV-1 replication in rodent cells has also been debated, although a reduction in the ratio of the unspliced 9-kb transcript to the fully-spliced 2-kb viral transcript in rodent cells has generally been reported [7,10]. Moreover, the role of the rat counterpart of hCRM1, which exports HIV RNAs in cooperation with Rev [20,21], has been incompletely explored. Instead, oversplicing or a reduced stability of unspliced transcripts in rodent cells compared to human cells has been proposed [22], which has been reported to be repaired by the expression of the human p32 protein [23].

In this study, we investigated the effect of human CyclinT1 and CRM1 expressed in rat T cells and macrophages, including primary cells, in order to identify a molecular basis for improving a rat model for HIV-1 infection. Our results show that co-expression of hCycT1 and hCRM1 synergistically promotes Gag p24 production. Interestingly, cell type specific requirements for these two human factors were detected.

## Methods

### Cells and plasmids

Rat T cell lines, FPM1 [25] and C58(NT)D (ATCC TIB-236), a rat macrophage line, NR8383 (ATCC CRL-2192), and human T cell lines, Jurkat and Molt4R5, were used for propagation of HIV-1. TZM-bl cells were used to measure the infectivity of HIV-1 according to previously described procedures [26]. NR8383hCRM1, FPM1hCRM1, FPM1hCT, and FPM1hCT/hCRM1 expressing hCRM1, hCycT1, or both were constructed as described previously [40].

To construct hemagglutinin (HA)-tagged hCycT1, pβCycT, which harbors the human cyclinT1 cDNA in the pCXN2 vector, was used as a template for PCR with forward (5'-ggtctagagcactatggagggagagaggaag-3') and reverse (5'-gggaattcatgcatagtctggtacatcgtaggggtacttaggaaggggtggaagtggtgg-3') primers with the following amplification conditions: 2 min at 94°C, 30 cycles of 30 s at 94°C, 60 s at 64°C, 2.5 min at 72°C, and a final extension for 10 min at 72°C. The amplified DNA was digested and inserted between the *Eco*RI and *Xba*I sites of pCXN2 [41].

Rat Cyclin T1 mRNA was extracted from rat ER-1 neo1 cells using the Absolute RNA extraction Kit (Stratagene) and amplified by RT-PCR using the following primers: 5'-ccgaattcaagcactatggagggagagaggaa-3' and 5'-ccgaattcatgcatagtctggtacatcgtaggggtacttaggaagaggtggaagaggtgg-3'. The amplification conditions were: 94°C for 2 min, 30 cycles of 15 s at 94°C, 30s at 60°C, 2.5 min at 68°C, and a final extension for 5 min at 68°C. The amplified DNA was digested and inserted into the *Eco*RI site of pCXN2.

To construct pSRαrCRM1-HA, pSRαrCRM1 was used for PCR with the following primers: 5'-ctggaatcacttggcagctgagctctacagagagagtcca-3' and 5'-tatggtaccttaagcataatcaggaacatcgtatgggtagtcacacatttcttctgggatttc-3'. The amplification conditions were: 2 min at 94°C, 20 cycles of 30 s at 94°C, 1 min at 62°C, 2 min at 68°C, and a final extension for 10 min at 68°C. The amplified DNA was digested and inserted into the SacI and KpnI sites of pSRαrCRM1.

The following plasmids were used in this study: pSRα296 [42]; pCRRE [35]; pΔpol [24]; pMaxGFP (Amaxa) and pCDMβ-gal [43]; pNL4-3 [30]; pYU-2 [28]; p89.6 [32]; pLAI-2 [31]; pYK-JRCSF [27]; and pNLAD8-EGFP [29]. pH1-luc (a gift from Dr. A. Adachi) contains a luciferase coding sequence downstream of the HIV-1 LTR. pSRαhCRM1-HA was a gift from Dr. T. Kimura.

### Development of Human Cyclin T1 Transgenic (Tg) Rats

An hCycT1 BAC (RZPD;RZPDB737F032099D) was microinjected into fertilized rat (F344) eggs. To identify Tg rats, total genomic DNA extracted from rat tail snips was examined by PCR using two sets of PCR primers with one primer annealing the BAC backbone vector and the other annealing the 5' or 3' end of hCyclin T1 genomic DNA. Primers CTB3 (gccaacgctcaatccggttctcgc) and CTGB3 (gctattttccagctgttctcgagtg) were used for the 5' end. Primers CTB4 (ttattccctagtccaaggatgac) and CTGB4 (cagacaatagactatcaagacactgtg) were used for the 3' end. PCR was performed using 500 ng of DNA as a template with the following amplification conditions: 94°C for 2 min, 30 cycles of denaturation (94°C for 1 min), annealing (58°C for the 5' end primers and 54°C for the 3' end primers, 30s), extension (72°C, 1 min), and a final extension (72°C, 5 min).

### Preparation of rat primary cells and human cells

Rat primary T cells were enriched from splenocytes using a nylon wool column. More than 95% of the cells were CD3^+ ^cells, as evaluated by Flow Cytometry (FACS Calibur; Becton Dickinson). The cells were stimulated for 2 days with an anti-rat CD3 mAb (5 μg/ml) and an anti-rat CD28 mAb (0.5 μg/ml) that had been coated on the culture plates. CD4^+^T cells were then isolated by negative selection using anti-rat CD8 MicroBeads (Miltenyi Biotec). Isolated CD4^+^CD8^- ^T cells were >90% pure, as determined by staining with anti-rat-CD4 (BD Biosciences Pharmingen) and anti-rat-CD8 (BD Biosciences Pharmingen).

Rat peritoneal macrophages were isolated from rats that had been treated with 3% thioglycollate for 3 days. The macrophages were coated with anti-rat CD11b and isolated using goat anti-mouse IgG MicroBeads (Miltenyi Biotec). Isolated CD11b^+ ^peritoneal cells were >90% pure, as determined by staining with mouse anti-rat-ED2 (BD Biosciences). Isolated CD11b^+ ^ED2^+ ^peritoneal cells were cultured for 2 h at 37°C to allow them to adhere to the plates.

Human peripheral blood mononuclear cells (PBMCs) were isolated from healthy donors using Ficoll Paque Plus (Amersham Biotechnology) density centrifugation. The cells were activated with 5 μg/ml phytohemagglutinin-P (PHA-P) (SIGMA) and 20 U/ml IL-2 (PeproTech EC) for 3 days at 37°C. Peripheral blood lymphocytes (hPBLs) were harvested as non-adherent cells.

Human monocytes were isolated from PBMCs using anti-CD14 conjugated to magnetic beads (Miltenyi Biotec), and allowed to adhere on dishes at 37°C for 1 h in RPMI 1640 supplemented with 1% human serum. Human monocyte-derived macrophages (MDMs) were then generated by incubation in RPMI 1640 supplemented with 15% FBS, antibiotics, and GM-CSF (10 U/ml) (R & D) for 5 days.

### Electroporation

Cell lines (2 × 10^6^) and primary T cells (1 × 10^7^) were electroporated in 100 μl of Nucleofector Solution (Cell line Solution V, Mouse T cell and human T cell Nucleofector kit, Amaxa Biosystems,) using the conditions (FPM1;T-03, C58(NT)D;T-20, NR8383;T-27, and rat primary T;X-01, Jurkat;X-01, Molt4R5;A-30, hPBL;U-14) and plasmids described in the Figure Legends. After 48 h, p24 in the supernatant and in cells was quantified using a p24 ELISA kit (Zeptometrix). In some cases, the viruses were concentrated by centrifugation at 15,000 rpm for 90 min in a microcentrifuge and p24 was quantitatively recovered from the pellets.

### Western Blotting

Cells were lysed in buffer containing 10 mM Tris-HCl, pH 7.4, 1 mM MgCl_2_, 0.5% NP40, and protease inhibitors or sample buffer without mercaptoethanol and dye, and protein concentrations were determined by BCA assay. Samples containing 50 μg protein were then subjected to Western blotting using anti-CycT1 (Novocastra Laboratories Ltd), anti-CRM1 [42], anti-HA (Behringer), or anti-β-actin (SIGMA).

### Infection

Rat peritoneal macrophages and human MDMs were seeded at a density of 5 × 10^5 ^cells/well in 24 well plates and cultured for 1 day at 37°C. Macrophages were then inoculated with VSV-G-coated NL43 and NLAD8-EGFP (50 ng), which were prepared by transfection of pNL4-3 or pNLAD8-EGFP along with pVSV-G to 293 T cells with Fugene6, in the absence or presence of 20 μM PMPA [44] overnight at 37°C. Finally, cells were washed gently 5 times and 2 ml of RPMI containing 15% FCS with or without PMPA was added.

## Results

### Synergistic Effects of hCycT1 and hCRM1 in Rat T cell lines

**Figure 1:**
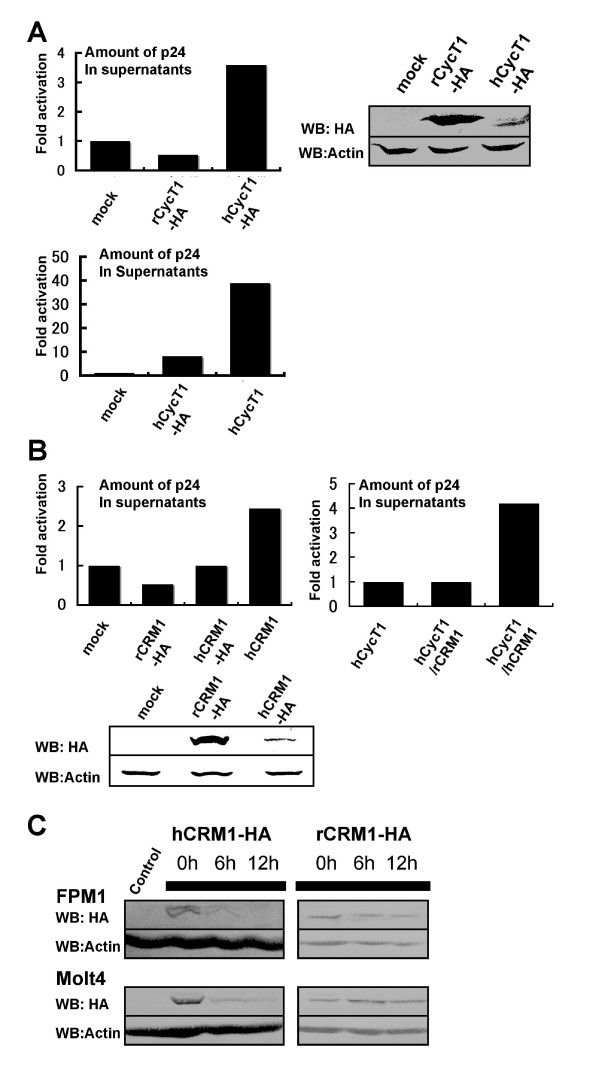
**Effect of hCycT1 and hCRM1 expression in rat T cell lines (part 1)**. (A) FPM1 cells were electroporated with 2 μg pΔpol, 1 μg pMax-GFP, and 1 μg pCXN2, pCXN2hCycT1-HA, pβhCycT1, or pCXN2rCycT1-HA. After 2 days, p24 levels in the medium were measured by ELISA. The percentage of living cells was approximately 18% and approximately 95% of the living cells were GFP^+ ^based on FACS analysis. The ratio of p24 in the CycT1 containing samples relative to mock treated samples was calculated. The total amount of p24 in the hCycT-HA containing sample was 119 pg. Values are means of duplicate samples. rCycT1 and hCycT1 were detected by Western blotting using anti-HA. (B) FPM1 cells were electroporated with 2 μg pΔpol, 1 μg pMax-GFP, and 0.5 μg pSRα296, pSRα hCRM1-HA, pSRαrCRM1-HA, or pSRαhCRM1. The percentage of living cells was approximately 4%, and 60% of the living cells were GFP^+^. The total amount of p24 in the sample containing hCRM1 was 146 pg. In the right panel, 1 μg pCNXhCycT1 was included. Values are means of duplicate samples. The total amount of p24 in the sample containing hCRM1 was 15.7 ng. (C) pSRα296, pSRαhCRM1-HA, or pSRαrCRM1-HA (0.5 μg) were electroporated into FPM1 and Molt4 cells, and 50 μg/ml cycloheximide was added after 24 h. The cells were then collected at 0, 6, and 12 h after the drug addition, and analyzed by Western blotting. Various amounts of the cell lysates were used for blotting (25 μg of hCRM1-HA containing FPM1, 5 μg rCRM1-HA containing FPM1, and 25 μg of hCRM1-HA or 10 μg of rCRM1-HA containing Molt4, respectively).

Since controversial results regarding the activity of Tat in rat cells have been reported, we compared the effect of hCycT1 versus rCycT1expression in rat T cells. To express the HIV-1 genome and CycT1 in rat T cells, we used the electroporation of CycT1 and an HIV-1 genome expressing plasmid, since we experienced very low rates of HIV-1 infection even with VSV-G coated particles. In our hands, electroporation was the only way to introduce enough HIV genome into rat T cells. We co-electroporated pMax-GFP or pCDM-βgal to monitor the efficiency of electroporation. When we electroporated pΔpol, which was constructed by deleting 328 base pairs in the pol gene of the infectious pNL43 genome [24], and HA-tagged hCycT1 or rCycT1 into FPM1 cells, a rat CD4^+ ^T cell line transformed with HTLV-1 [25], Gag p24 production was enhanced several fold in the presence of hCycT1-HA. However, hCycT1 expression was very low. In contrast, rCycT1-HA was efficiently expressed, but did not alter Gag p24 production. Since hCycT1-HA may be unstable, we next used an untagged hCycT1 for co-electroporation. We detected a 40 fold enhancement of Gag production in the presence of hCycT1 (Fig. 1A). The band corresponding to hCycT1 was, however, hardly detected by Western blot analysis (data not shown). The reason why untagged hCycT1 enhanced expression more efficiently than hCycT1-HA is currently unclear, because the intracellular amounts of these hCycT1s cannot be exactly compared due to the different abilities of the anti-HA mAb and anti-hCycT1 antibody.

Next, to assess Rev activity in rat T cells, we compared the effects of hCRM1 and rCRM1 on HIV-1 propagation. When we electroporated HA-tagged CRM1 expression plasmids and pΔpol into FPM1 cells, p24 production was not significantly increased. The level of hCRM1-HA detected by Western blotting was very low. However, we reproducibly observed a 2–4 fold enhancement of p24 production in cells transiently expressing untagged hCRM1, but not rCRM1 (Fig. 1B). These results suggest that endogenous rCRM1 supports p24 production less efficiently than the hCRM1 and that Rev function is not absolutely blocked in rat T cells. To examine the stability of CRM1-HA, we added cycloheximide to inhibit translation in CRM1-transfected T cells and examined CRM1 protein levels over time. In both rat and human T cells, hCRM1-HA was much less stable than rCRM1-HA (Fig. 1C), partly accounting for the lower amounts of hCRM1 (See Fig. 1B).

**Figure 2:**
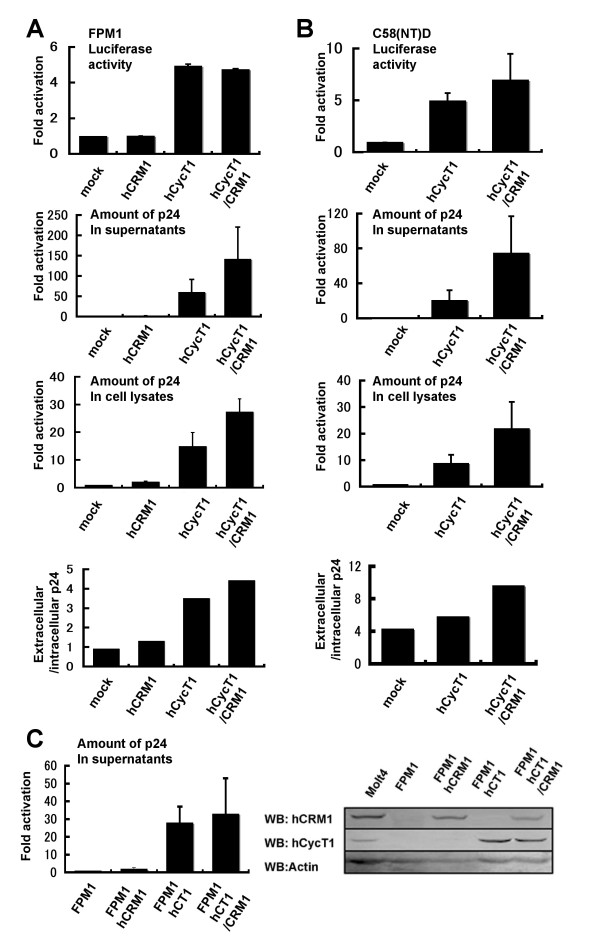
**Effect of hCycT1 and hCRM1 expression in rat T cell lines (part 2)**. (A) FPM1 and (B) C58(NT)D cells were electroporated, as above, with the exception that 0.4 μg pH1-Luc and 0.2 μg pCDMβ-gal were used instead of pMax-GFP. LTR activity and transfection efficiency were measured by luciferase and β-gal assays using cell lysates. The luciferase/β-gal activity or the amount of p24 was calculated, and the value of the mock sample was normalized to 1. Values are means of triplicate samples and the SD was calculated. The amount of p24 in the FPM1 and C58(NT)D samples containing hCycT1/hCRM1 was 3.7 and 2.8 ng, respectively. (C) FPM1 cells continuously expressing hCycT1 and hCRM1 were electroporated with 4 μg pNL4-3 and 1 μg pMaxGFP. The percentage of living cells was approximately 10%, and 50% of the living cells were GFP^+^. The amount of p24 in the FPM1hCT/hCRM1 sample was 6.0 ng. Approximately 10 μg of each cell lysate were subjected to Western blotting.

To examine the effects of both hCycT1 and hCRM1 on HIV-1 propagation in rat T cells, including FPM1 and C58(NT)D cells, we co-electroporated these expression plasmids with pΔpol. Additionally, we co-transfected pH1-Luc, which expresses the luciferase gene driven by the HIV-1 LTR, to examine the effect of hCycT1 and hCRM1 on Tat-directed gene expression. Expression of hCycT1, but not hCRM1, enhanced LTR-derived expression several fold, consistent with the previously reported functions of these proteins. Notably, the enhancement of p24 production by hCycT1 was substantially greater than that of the luciferase activity. Furthermore, levels of extracellular p24 were more enriched than intracellular levels, and hCycT1 synergistically cooperated with hCRM1 to augment the synthesis of p24 by approximately 100 fold (Fig. 2A and 2B). These results suggest that hCycT1 enhanced the transcription of the LTR-driven HIV-1 pre-mRNA. Since the pre-mRNA is the source of mRNAs encoding Gag, Tat and Rev, its increase may trigger positive feedback in the synthesis of HIV-1 pre-mRNA as a result of increased Tat protein levels and in the amounts of unspliced mRNA as a result of increased Rev protein levels. Thus, Gag would be produced much more efficiently than luciferase. Subsequently, the enhanced Gag expression facilitates the more efficient release of viral particles. The level of p24 produced by rat T cells expressing both hCycT1 and hCRM1 was approximately 25–33% of the levels produced by the human T cell line Molt4 (data not shown).

To examine the effect of hCycT1 and hCRM1 on HIV-1 propagation using a full length HIV-1 clone, we electroporated pNL4-3 into FPM1 T cells that continuously expressed hCycT1 and hCRM1, and then quantified the production of p24. Again, hCycT1 greatly augmented p24 production, and hCRM1 had a moderate effect. Notably, the levels of hCycT1 and hCRM1 expression in FPM1 cells were similar to those in Molt4 cells (Fig. 2C). Thus, expression of these human factors should support robust HIV-1 propagation in rat T cells.

### Synergistic Effects of hCycT1 and hCRM1 in rat macrophages

**Figure 3:**
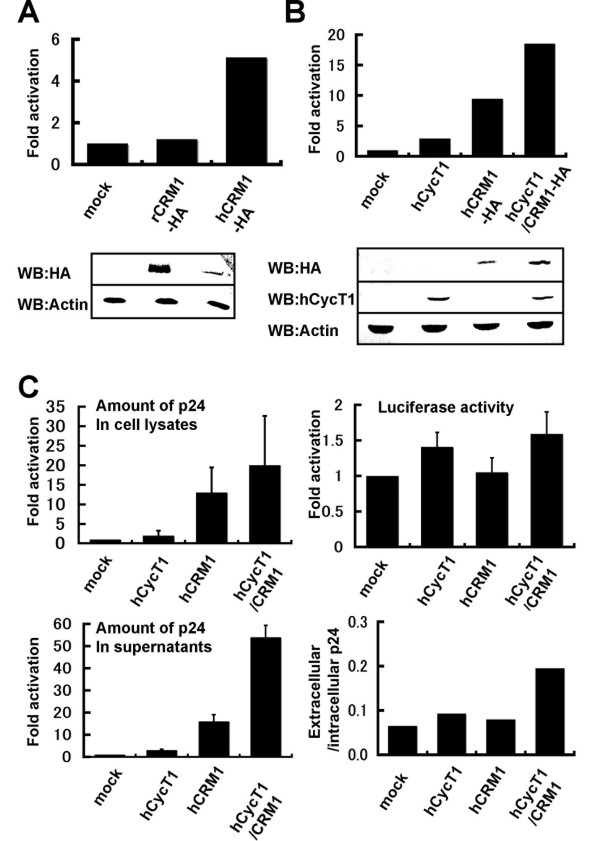
**Synergistic effect of hCycT1 and hCRM1 in rat macrophage cell lines**. (A) NR8383 cells were electroporated as described in Fig. 1B. The percentage of living cells was approximately 20–40%, and approximately 75% of the living cells were GFP^+^. The amount of p24 in the sample containing hCRM1-HA was 196 pg. Approximately 50 μg samples of the cell lysates were subjected to Western blotting as described in the Methods. (B) NR8383 cell lines were electroporated as described in Fig. 1A. The percentage of living cells was approximately 15%, and approximately 60% of the living cells were GFP^+^. The amount of p24 in the sample containing hCRM1-HA/hCycT1 was 56 pg. (C) NR8383 cell lines were electroporated with 2 μg pΔpol, 0.4 μg pH1-Luc and 0.2 μg pCDMβ-gal along with or without 1 μg pβhCycT1 and 0.5 μg pSRαhCRM. pSRα296 was added to adjust the total amount of the plasmids. The amounts of p24 in the cell lysate and medium of the sample containing hCRM1/hCycT1 were 488 and 96 pg, respectively. Values are means of triplicate samples.

We examined the effect of hCycT1 and hCRM1 on p24 production and LTR-driven expression in the rat macrophage cell line NR8383, using the experimental approaches described above. Transient expression of rCRM1-HA in NR8383 cells did not affect p24 production, whereas hCRM1-HA enhanced p24 production 5–10 fold, although the level of hCRM1-HA expression was much less than that of rCRM1-HA (Fig. 3A). Expression of hCycT1 enhanced p24 production by only a few fold. The expression of hCycT1 was readily detected by Western blotting (Fig. 3B), in contrast to the low levels in rat T cells. Neither hCycT1 nor hCRM1 expression significantly affected luciferase expression driven by the HIV LTR (Fig. 3C). We also detected a greater than 10 fold enhancement of extracellular and intracellular p24 production in the presence of untagged hCRM1 (Fig. 3C), but not rCRM1 (data not shown). When hCycT1 and hCRM1 were co-expressed, they synergistically augmented p24 production by greater than 20–50 fold in NR8383 cells (Fig. 3B and 3C). The amount of extracellular p24 increased more than intracellular p24, as seen in T cells, suggesting that the increase in Gag expression facilitated more efficient release of viral particles. These results clearly indicate that hCRM1 augments p24 production in rat macrophages more efficiently than hCycT1, in contrast to the effects of the two proteins in rat T cell lines.

### Infectivity of HIV-1 produced by rat cells

**Figure 4:**
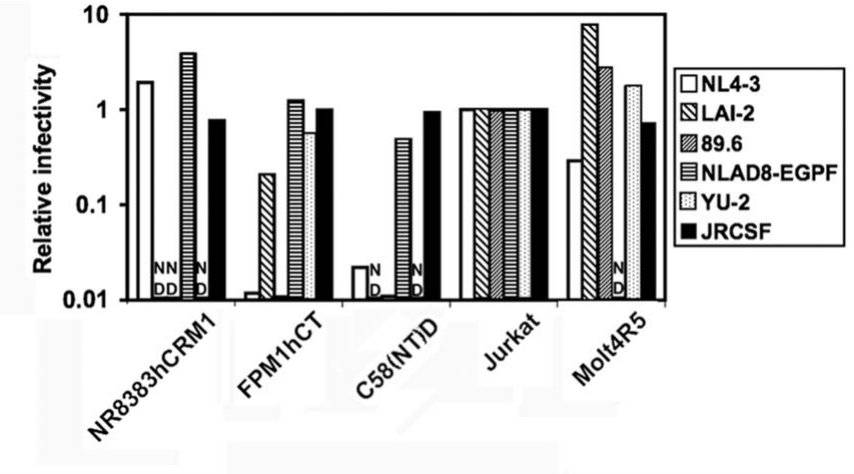
**Infectivity of HIV-1 produced in rat and human cells**. The medium [containing 50 or 500 pg of p24] from the various cell types electroporated with infectious clones was used to infect TZM-bl cells, and luciferase activity in the TZM-bl cells infected with various progeny viruses was normalized to that in cells infected with HIV-1 released from Jurkat cells. The relative infectivity of HIV-1 from Jurkat cells was normalized to 1. N.D: not determined.

To investigate whether HIV-1 produced by rat cells is infectious, we electroporated infectious HIV-1 molecular clones into rat and human cells and evaluated the infectivity of the progeny viruses using the indicator TZM-bl cells, which express luciferase upon HIV infection [26]. Luciferase activity versus inoculated p24 was used as a surrogate marker of infectivity. Interestingly, R5 viruses produced in rat T cells, including the JR-CSF [27], YU-2 [28], and NL-AD8 [29] strains, were equally infectious compared to those produced by human T cells, whereas rat T cell-derived ×4 and dual tropic viruses such as NL4-3 [30], LAI-2 [31], and 89.6 [32] varied in their infectivity. In contrast, both R5 and ×4 viruses produced in the macrophage cell line exhibited infectivities comparable to those from human cells (Fig. 4).

### Characterization of hCycT1 and hCRM1 Tg rats

**Figure 5:**
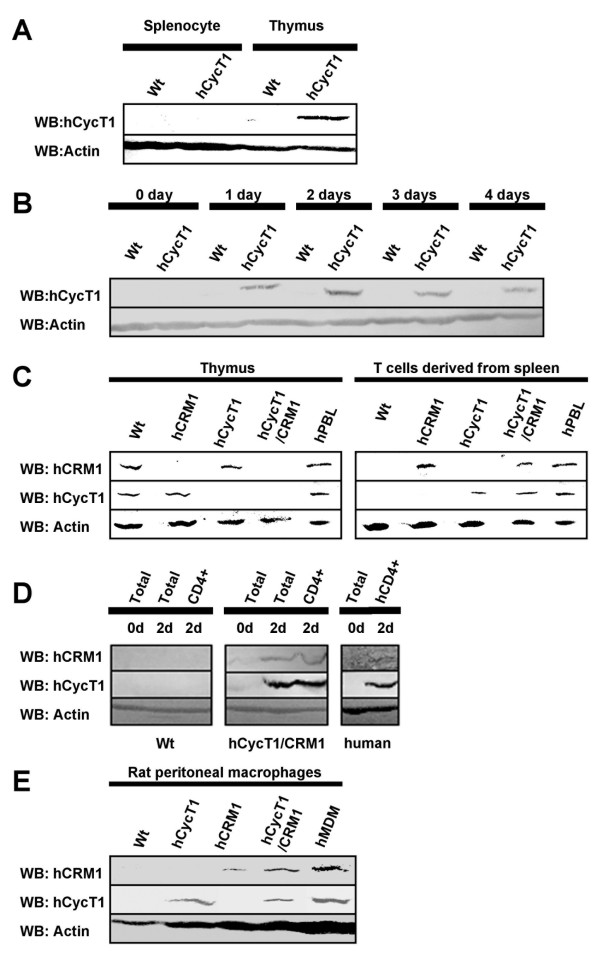
**Characterization of hCycT1 and hCRM1 Tg rats**. (A) The expression of hCycT1 in spleen- and thymus-derived cells from WT or hCycT1 Tg rats was confirmed by Western blotting using anti-hCycT1. (B) T cells derived from the spleen of WT or hCycT1 Tg rats were stimulated with anti-rat-CD3 and anti-rat-CD28. Cells were collected at the indicated times and subjected to Western blotting using anti-hCycT1. (C) The expression of hCycT1 and hCRM1 in spleen- and thymus-derived cells (C), total T and CD4^+^CD8^- ^T cells (D), and macrophages (E) in WT or Tg rats was confirmed by Western blotting using anti-hCycT1 and anti-hCRM1. T cells derived from the spleen of WT or hCycT1 Tg rats were stimulated with anti-rat-CD3 and anti-rat-CD28.

To examine the role of hCycT1 in primary cells, we constructed transgenic (Tg) rats that express hCycT1. Since the regulation of cyclinT1 gene expression is complex [33], a BAC harboring the entire human cyclinT1 gene, which is assumed to contain all the regulatory sequences, was microinjected into fertilized rat eggs. To confirm the expression of hCycT1 in the Tg rats, cells isolated from both thymus and spleen were analyzed by Western blotting using anti-hCycT1. Thymocytes, but not splenocytes, of Tg rats expressed hCycT1 (Fig. 5A). Since hCycT1 is expressed during the activation of human lymphocytes [33], we stimulated the splenocytes with anti-CD3 and anti-CD28. Expression of hCycT1 was detected within 1 day and peaked 2 days after stimulation (Fig. 5B). Interestingly, rat splenocytes stimulated with phytohemaglutinin (PHA) and IL-2 did not express hCyCT1 (data not shown).

Expression of hCRM1 in Tg rats was also examined, using a previously established Tg rat [34]. hCRM1 was expressed in both thymocytes and splenocytes activated with anti-CD3/CD28 (Fig. 5C). hCRM1 was not expressed in unstimulated splenocytes (data not shown), consistent with hCRM1 expression in human PBMC [34]. We further characterized total T cells and CD4^+^CD8^- ^T cells prepared from double Tg rats in comparison to rat total T cells and human CD4^+^CD8^- ^T cells 2 days after stimulation. Both hCycT1 and hCRM1 were expressed in activated CD4^+^CD8^- ^T cells prepared from the Tg rat, similar to human CD4^+^CD8^- ^T cells (Fig. 5C and 5D). Both hCycT1 and hCRM1 were expressed in rat peritoneal macrophages at levels equivalent to expression in human monocyte-derived macrophages (MDMs) (Fig. 5E).

### Ex vivo p24 production in T cells derived from hCycT1/CRM1 Tg rats

To investigate the effects of hCycT1 and hCRM1 on p24 production in primary T cells, we prepared T cells from splenocytes of wild-type (WT) and Tg rats and stimulated them with anti-CD3/CD28. As a control, isolated human PBLs were activated. In these experiments we used pCRRE [35], which harbors an HIV-1 genome with a deletion in the region from pol to vpr, instead of pΔpol [24], since introducing either pΔpol or the full-sized HIV-1 genome into the primary T cells by any method, including electroporation or VSV-G coated virus, had limited success.

**Figure 6:**
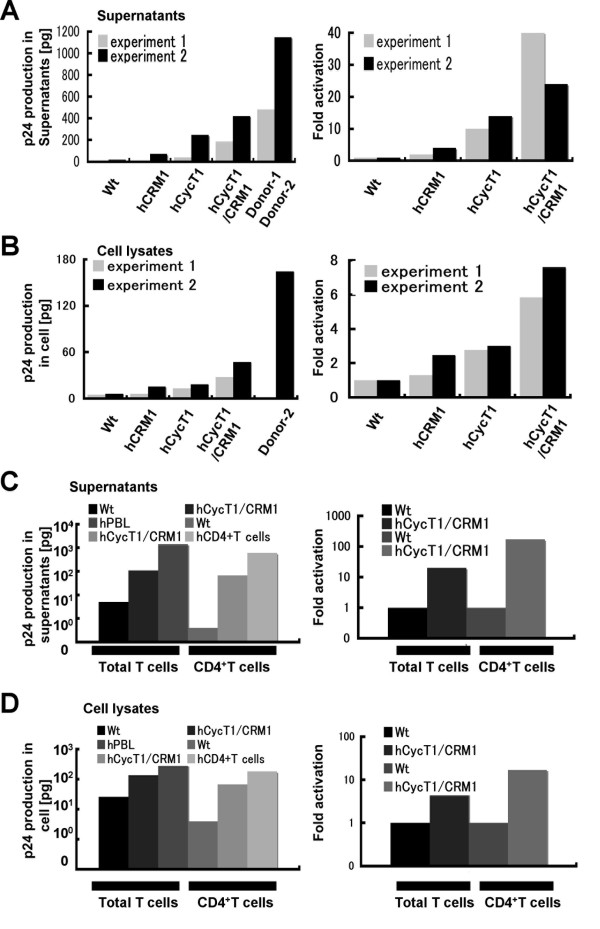
**Quantification of p24 production in the total T cell fraction and CD4^+^CD8^- ^T cell fraction derived from hCycT1/CRM1 Tg rats**. Stimulated spleen-derived T cells from WT or Tg rats and hPBL were electroporated with 4 μg PCRRE and 1 μg pMax-GFP, and p24 production in the supernatants (A) and cell lysates (B) was measured by ELISA (left panel). The percentage of living cells was 30–40%, and 28–40% of the living cells were GFP^+^. The right panels represent the fold activation of Tg versus WT rats. Stimulated CD4^+^CD8^- ^T cells derived from WT, hCycT1/CRM1 Tg rats, and human blood were electroporated, as above, and p24 production in the supernatants (C) and cell lysates (D) was measured. The percentage of living cells was ~10%, and 30–40% of the living cells were GFP^+^. Values are the means of duplicate samples.

T cells derived from hCycT1 Tg rats produced approximately 10–15 fold more p24 than WT T cells. In T cells derived from hCRM1 Tg rats, p24 production increased approximately 3 fold over WT cells. T cells-derived from hCycT1/CRM1 doubly Tg rats produced p24 at levels 24–40 fold greater than WT, and this level was ~40% of that produced by hPBLs (Fig. 6A). We further examined p24 production by CD4^+^CD8^- ^T cells prepared from double Tg rats in comparison to WT rat and human cells. CD4^+^CD8^- ^T cells prepared from double Tg rats produced p24 in the medium approximately 180 fold more efficiently than WT rat cells; this level was ~11% of the amount of p24 produced by human CD4^+^CD8^- ^T cells (Fig. 6C). These results indicate that the synergistic effects of hCycT1 and hCRM1 promoted the production of p24 in rat primary T cells *ex vivo*.

When intracellular p24 was evaluated by ELISA, increases of approximately 7 and 17 fold were observed in total T and CD4^+^CD8^- ^T cells, respectively (Fig. 6B and 6D), considerably less than the amount of extracellular p24 described above. The ratio of extracellular p24 to intracellular p24 increased gradually as p24 production increased, suggesting a more efficient virus release from the double Tg rat T cells compared to WT rat T cells.

### Ex vivo p24 production in peritoneal macrophages derived from hCycT1/CRM1 Tg rats

**Figure 7:**
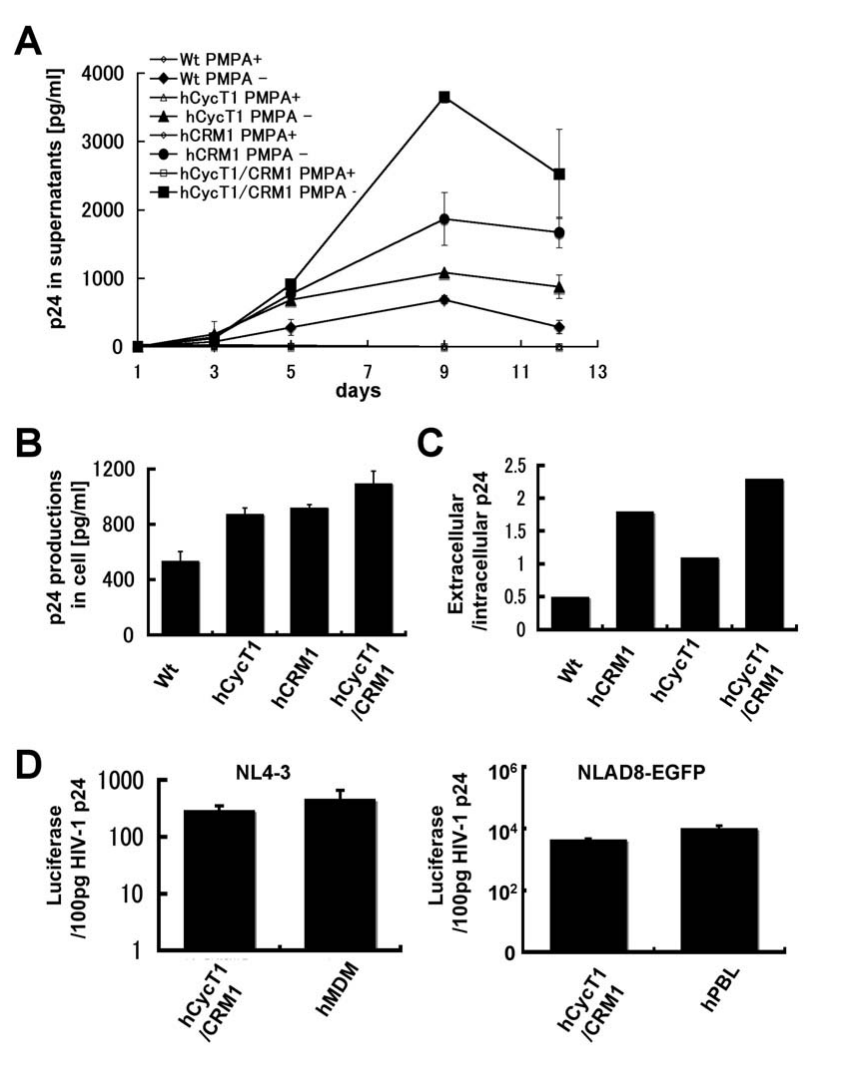
**Quantification of p24 production in rat peritoneal macrophages**. (A) Rat peritoneal macrophages or human MDMs were infected with VSV-G pseudotyped NL4-3 virus. The amount of p24 in the medium was then measured by ELISA. (B) The infected cells were harvested 12 days after infection and intracellular p24 levels were evaluated. (C) The ratio of the amount of extracellular to intracellular p24 was calculated. (D) Infectivity of viruses present in the medium 5 days after infection was measured using TZM-bl cells. NLAD8-EGFP was used to infect 5 × 10^5 ^macrophages from double Tg rats or human PBL, and the medium was recovered 5 days after infection. Values are the means of triplicate samples.

To investigate HIV-1 propagation in macrophages derived from Tg rats, we prepared CD11b^+^ED2^+ ^peritoneal macrophages and subsequently infected the cells using HIV-1 pseudotyped with VSV G protein. Although WT peritoneal macrophages produced a considerable amount of HIV-1 progeny virus in the absence of hCRM1 and hCycT1 expression, macrophages derived from hCycT1/CRM1 doubly Tg rats produced 6 fold higher levels of p24 at their peak (Fig. 7A). This level corresponds to 20% of the amount of p24 produced by human MDMs (data not shown). Macrophages from hCRM1 Tg rats supported a several fold increase in p24 production, but hCycT1 expression had a smaller effect. Macrophages treated with PMPA, a reverse transcriptase inhibitor, did not produce significant amounts of p24, confirming that the p24 measured represents production of progeny viruses and not inoculum. The amount of intracellular p24 also increased to some extent in the Tg rats, but to a lesser extent than p24 levels in the medium (Fig. 7B). Approximately 67% of the p24 synthesized in the doubly Tg cells was released into the medium and the ratio of extracellular p24 to intracellular p24 increased as viral production increased (Fig. 7C).

The infectivity of the viruses, which were harvested 5 days post infection, was evaluated using TZM-bl cells. Figure 7D shows that both R5 and ×4 viruses produced from rat macrophages retained infectivity levels similar to those from human PBLs and MDMs.

## Discussion

In the present study, we demonstrated the effects of hCycT1 and hCRM1 on augmentation of HIV-1 Gag production in both established and primary rat T cells and macrophages. hCycT1 enhanced p24 production profoundly in rat T cells, suggesting that hCycT1 is an essential gene that should be included in the construction of a rat model of HIV-1 infection. Although our results are in contrast to the previous reports of only a 2–5 fold increase in early gene expression in rat primary T cells and epithelial cells expressing hCycT1 [7,10,16,17], the overall effects stemmed from the increased HIV-1 pre-mRNA in response to hCycT1 expression included an increase in Tat/Rev proteins and enhanced efficiency of p24 release from T cells. This may explain the remarkable enhancement of p24 levels in the extracellular milieu. Our results support and extend the effect of hCycT1 expressed in rat primary T cells originally described by Michel et al [17]. In contrast, hCycT1 expression in macrophages had only a minor effect on p24 production. Since the level of LTR-driven luciferase activity in NR8383 cells in the absence of hCycT1 was similar to Molt4 cells (data not shown), the high basal activity of LTR-driven gene expression may explain the diminished effect of hCycT1 expression. These data are consistent with the relatively high HIV-1 LTR activity in primary macrophages [7,16,17]. Since rCycT1, like mCycT1, has a tyrosine at residue 261 in place of the hCycT1 cysteine [7], which is crucial for binding to the TAR element, rCycT1 itself may not be functional in LTR-driven expression. Instead, rat epithelial cells and macrophages may support transcription in a Tat independent manner. Alternatively, other factors in these cells may cooperate with rCycT1 for efficient LTR-driven expression.

The expression of hCRM1 in the rat macrophage line NR8383 profoundly augmented the production of p24, suggesting that Rev function is impaired and that inclusion of the hCRM1 gene in construction of a rat model for HIV-1 infection should be considered. Moreover, the profound effects of hCRM1 expression have been observed in several rat epithelial cell lines (data not shown); rCRM1 may support Rev function less efficiently. However, the effect of hCRM1 was not as great in T cell lines, primary T cells, or macrophages, compared to the macrophage cell line. These observations suggest that CRM1 function may be affected by factors involved in the formation of gag mRNA, such as the cell type-specific efficiency of splicing.

In mouse cells, defects in HIV particle formation and release have been reported [11] due to incorrect transport of gag mRNA from the nucleus to the cytoplasm [36]. The release of viral particles from both primary rat T cells and macrophages was inefficient when p24 production was low. However, when p24 production was enhanced by expression of hCycT1 in T cells or hCRM1/hCycT1 in macrophages, p24 was released more efficiently. These results suggest that the intracellular concentration of Gag protein is critical for efficient virus formation. However, rat tetherin, which is resistant to Vpu-induced degradation, may reduce the release of viral particles, although this effect was demonstrated using tetherin overexpression [37]. Since we observed that the efficiency of viral release was variable under different conditions (compare panels of Fig. 6), the inhibitory effect of rat tetherin may be an important subject for future study.

Both R5 and ×4 viruses produced from rat macrophages are as infectious as those produced by human macrophages, consistent with the report of Keppler et al. [8]. In contrast, ×4 and dual-tropic viruses that were produced in rat T cells had varying infectivities, although several R5 strains produced in rat T cells were as infectious as human T cell-produced viruses. These differences in infectivity may be ascribed to the envelope because the AD8 strain was constructed by substituting M-tropic *env *for the counterpart *env *fragment in pNL4-3 [29]. Investigating the causes of these differences in infectivity will enable us to make a rat model that allows for propagation of various strains of HIV-1.

The efficiency of the early steps of infection, including reverse transcription, nuclear import, and integration in macrophages and T cells of Sprague-Dawley rats is comparable to human cells, in contrast to the low rate of integration in mouse T cells [8,16,38]. We have also efficiently infected rat macrophages using VSV-G-coated viruses. However, the very low rate of infection of primary T cells from the rat F344 strain used in this study has hampered our detailed analysis, and suggested that inhibitory factors affecting viral penetration, similar to monkey Trim5α [39], may be present. Further studies on the mode of HIV infection in each rat strain will be required.

## Conclusion

Expression of both hCycT1 and hCRM1 synergistically enhanced p24 production in rat T cells and macrophages to levels approximately 10–40% of those detected in human cells. R5 viruses produced in the rat cells were infectious. Moreover, the efficiency of the early steps of HIV-1 infection in some rat cells has been reported to be comparable to human cells [8,16]. Collectively, these results suggest that rats that express human CD4, CCR5, CycT1, and CRM1 may provide the basis for a good model system that supports multiple cycles of HIV-1 infection.

## Competing interests

The authors declare that they have no competing interests.

## Authors' contributions

HS and TO designed the study. HO conducted the majority of the experiments. XZ performed and analyzed infection experiments. IBF and HS constructed and maintained the transgenic rats. MN constructed HA-tagged CRM1 plasmids. HS and HO wrote the paper. All authors approved the final manuscript.
